# Prion Strains and Transmission Barrier Phenomena

**DOI:** 10.3390/pathogens7010005

**Published:** 2018-01-01

**Authors:** Angélique Igel-Egalon, Vincent Béringue, Human Rezaei, Pierre Sibille

**Affiliations:** Virologie et Immunologie Moléculaires, INRA, Université Paris-Saclay, UR892, 78350 Jouy-en-Josas, France; Angelique.egalon@inra.fr (A.I.-E.); vincent.beringue@inra.fr (V.B.); human.rezaei@inra.fr (H.R.)

**Keywords:** prion strain, species barrier, strain adaptation, zoonosis, Darwinian evolution, deformed templating, structural elementary brick

## Abstract

Several experimental evidences show that prions are non-conventional pathogens, which physical support consists only in proteins. This finding raised questions regarding the observed prion strain-to-strain variations and the species barrier that happened to be crossed with dramatic consequences on human health and veterinary policies during the last 3 decades. This review presents a focus on a few advances in the field of prion structure and prion strains characterization: from the historical approaches that allowed the concept of prion strains to emerge, to the last results demonstrating that a prion strain may in fact be a combination of a few quasi species with subtle biophysical specificities. Then, we will focus on the current knowledge on the factors that impact species barrier strength and species barrier crossing. Finally, we present probable scenarios on how the interaction of strain properties with host characteristics may account for differential selection of new conformer variants and eventually species barrier crossing.

## 1. Introduction

Scientists have been intensively working on prion diseases, nevertheless, several aspects of this transmissible neurodegenerative affection remain obscure. Among these black boxes, the biophysical support for prion strain variation and the species barrier have stand as one of the last accessible achievements. Although being known for a long time, scrapie and related diseases really went in the light in the 90’s: society urged scientists for answers to the questions raised by the sudden outbreak of mad cow disease (caused by Bovine Spongiform Encephalopathy (BSE) prions), which could result in a Creutzfeldt-Jakob Disease (CJD)—like disease in humans. At that time, the potential transmission of BSE from cattle to humans had been assessed persistently, leading to perplexing and even opposite results (sometimes coming from the very same lab) [1,2]. Rather than the consequence of an urgent need for results, these inconsistent data underlined the complexity of the addressed question that we are still trying to answer for the last 30 years. This review aims at presenting the main knowledge and the latest milestones in the field of prion strains and species barrier phenomena, both topics being intimately linked.

Prion diseases are fatal neurodegenerative disorders. At the beginning of the 20th century, Drs Creutzfeldt and Jakob first described the pathology in humans as a sporadic disease. Then the disease was identified as the main responsible for an epidemic of neurodegenerative cases among the Fore population of Papua New Guinea, leading the scientists (Gajdusek, Gibbs and Alpers) to propose an infectious spread of the disease (due to endocannibalistic rituals) [3]. Soon after, Griffith proposed a self-replicating model for the related scrapie disease [4] but the formal conceptualization of prion as a protein only infectious agent, responsible for the misfolding of the host cellular prion protein into a pathologic conformer had to wait for Prusiner’s seminal work (1982) [5]. The prevalence of the disease in human population is rather low (~1 case per million people per year, mostly among aged population). Some cases (~15%) are genetically linked, due to point mutation in the prion protein gene (*PRNP*). This disease is also long known to affect ruminants, including sheep and goats with scrapie, cattle with bovine spongiform encephalopathy and cervids with chronic wasting diseases (CWD). No crossing of the species barrier between human and ruminant prions have been reported until the implementation of new biophysical parameters in the process for recycling the livestock carcasses into the ruminant alimentary chain: these modifications resulted in incomplete inactivation of the BSE prions and paved the way for this unconventional agent to cross barrier species and to spread in humans in an outbreak known as the mad cow disease and the variant CJD (vCJD), respectively. And prompted scientists to further study the propensity of this agent to adapt from one host to the other.

## 2. Experimental Prion Transmission

### 2.1. Early Cases of Prion Interspecies Transmission

The first report on experimental prion transmission in animals focused on reproducing disease-specific clinical signs by inoculating infected brain extract [6]. The studied parameters were the incubation time until disease end stage, the nature of the clinical signs and the anatomic distribution of the lesions that were reported on a score profile. The first experiments have been reported in sheep [7]. Then, Pattison and colleagues reported several successful experimental inoculations of sheep scrapie to goats [8,9]. By that time, it was found that some prions could pass from one species to the other (e.g., mink to small ruminants [10]). As susceptible to infection with most prion strains, the bank vole turned out to act like a “universal acceptor” [11,12,13] (see also Section 4.1 and Section 4.2). Conversely, several studies reported on the difficulty to pass prions from one species to others (e.g., certain scrapie isolates to cattle [14]). 

With the development of transgenic mouse engineering, expression of foreign PrP (in the presence or in the absence of endogenous mouse PrP) considerably enlarged the possibilities for studying zoonotic transmission of prions. These approaches proved to be versatile, since it was demonstrated that development of prion pathology relied solely on the presence of convertible PrP [1,15,16,17]. In many cases, these experimental setups made emerge the idea that almost every prion could adapt to almost every PrP substrate, provided that some critical parameters (presented below) have been set up in order to adapt the strain to its new host PrP.

### 2.2. Emergence of the Prion Strain Concept

Interestingly they also noticed some reproducibility of the observed clinical signs, depending on the inoculated isolate. The conclusion of their papers proposed that “certain “strains” of the scrapie agent will produce the nervous syndrome, while others will produce the scratching syndrome”. Soon in the prion scientific history appeared the fact that prion agents could share common strain features with conventional DNA-encoded pathogens. These prion strain features could be distinguished from each other based on a number of parameters such as incubation time and titration, which were remarkably reproducible among strains in a given host. Addition of anatomical data such as the localization and intensity of vacuolation allowed to isolate and further characterize prion strains, that were secondarily used to study their adaptation when passing from a host to the other [18]. With the refinements in biochemical and biophysical analysis methods, several parameters are now available for the extensive study on prions that will be detailed in the following sections.

## 3. From Prion Strain Characterization

### 3.1. First Approaches

#### 3.1.1. Incubation Time, Clinical Signs, Vacuolation Tissue Tropism

Prion diseases are first characterized by clinical signs observed on the affected individuals. In humans, after a long pre-clinical period is ended, affected individuals usually complain with vague sensory feelings, such as depression. Progressive motor paralysis and dementia then rapidly follow. Cerebellar ataxia is often found in the course of the disease [19]. In animals, clinical signs include progressive ataxia as well but some features like itching and scratching seem to be animal-specific. Behaviour modifications, including aggressiveness or enhanced tameness are also recorded [9]. But these general features are markedly influenced by the strain of prion infecting individuals [8,20]. Distribution and abundance of the lesions in specific brain areas, which appeared to be remarkably stable, were used to score and attribute a “lesion” profile to prion strains [16]. Incubation period for a given strain in a given host was highly reproducible and rapidly served as the first criterion for the characterization of prion strains [21]: for instance, 263 K strain kills golden hamster in 65 days, whereas 139H strain kills the same species in 130 days (after intracranial inoculation). It is worth mentioning that inoculum dilution influences the incubation time [22,23]. Thus, a combination of criterions is necessary for efficient discrimination.

Immunoreactivity of antibodies reacting with the diseased form of the PrP (PrP^Sc^) allowed for significant refinement in the prion strain characterization [20]. Although prions ultimately accumulate in the Central Nervous System (CNS) of their host, peripheral accumulation may also be associated with some prion strains, contrasting with the ones that are only detected in the brain: in this respect, the most relevant tissues turned out to be the secondary lymphoid organs such as lymph nodes and spleen [24]. This lymphotropism may be used for discrimination between two strains that otherwise would look identical in the brain tissue [25].

#### 3.1.2. Biophysical Parameters (Circular Dichroism, Infra Red, …)

Several biophysical approaches have been employed for elucidating the parameters underneath the biological strain phenomenon. Due to its major insolubility and to the highly heterogeneous aspects of the prion material, all conventional approaches (X-ray crystallography, nuclear magnetic resonance (NMR)) mainly revealed unsuccessful in providing good quality crystals or homogenous solutions for the determination of a pathological PrP^Sc^ structure. Though, infra-red approaches first identified strain differences [26]: absorption spectra from Hyper, Drowsy and 263 K hamster strains revealed a difference between the Drowsy strain and the two others. These results were interpreted as differences in β-sheet secondary structures. Using a combination of several investigation techniques, the structural properties of two sorts of fibrils formed under different experimental conditions was further shown to correspond to differential folding patterns of β strands [27]. NMR imaging proved limited in exploring the PrP^Sc^ structure because of insolubility matters. Conversely, Hydrogen/Deuterium exchange proved helpful with solid NMR approaches [28]. Mass spectrometry using acetylation of accessible lysines in PrP^Sc^ assemblies recently added some arguments in favour with a β-solenoid form of the pathologic prion structure [29]. In addition, the approach was able to reveal structural differences between several common prion strains. Notably, Sc237 hamster prion strain is supposed to have an N-terminal fragment reacting less with the core prion protein than the others hamster strains tested. This view is further supported by the fact that Sc237 infectivity is less sensitive to PK-digestion (partial resistance of the PrP^Sc^ protease digestion is used as the gold standard for the detection of infected samples) than the others.

#### 3.1.3. Biochemical Methods (Western Blot, Resistance to Chaotropic Agents, Conformation-Dependent Immunoassays…)

At first, proteinase-K resistance of the pathological PrP^Sc^ form served as a diagnostic tool, since most of the antibodies raised against the PrP could not discriminate between normal PrP^C^ and pathological PrP^Sc^ forms of the prion protein in western blot. The PrP^Sc^ isoform partially resists to the cleavage by the most common proteases, among which proteinase K (PK): three major conformation have been reported according to the protease-resistant core size observed by western blotting: 21 kDa (type-1), 19 kDa (type-2) or 8 kDa with some specific antibodies specially recognizing some of these types such as 12B2, a type 1-specific antibody [30]. This distribution of prion types is however generally not exclusive: several CJD cases are actually a mix of T1 and T2 [31]. Whether this co-occurrence aroused from biochemical reasons or just by chance remains to be addressed.

The size of the resistant-core depends on the prion strain and its evaluation still remains the gold standard of prion analysis. Antigenic epitope mapping of PrP^Sc^ raised against different prion strains showed specific immunoreactivity [32], indicating conformational differences within PrP^Sc^ assemblies. Even if several monoclonal antibodies (mAbs) have been raised against PrP antigens from various species, in most cases however, mAbs were poor discriminants and cross reactivities were often recorded between mouse, hamster, human and most common ruminants, as it is the case for the conformational antibody 15B3 [33]. What’s more, these mAbs would hardly discriminate between normal and pathological PrPs, which would have been of enormous interest for diagnostic or laboratory purposes.

In addition to providing antibodies more or less selective for a given PrP protein, alternative approaches were designed for the study and conformational screening of prion strains [34]. Known as the conformation-dependent immunoassay (CDI), this technique depends on differential recognition of unmodified PrP^Sc^ or altered PrP^Sc^ epitopes to determine a ratio that can be used for direct quantitation of prion in a sample. This technique shows that more than 90% of PrP^Sc^ present in sCJD patients are PK-sensitive [35]; moreover, this ratio may reveal variations from strain to strain, as observed for eight characterized hamster-prion strains [34]. In particular, this approach allowed for a better discrimination between prion strains that otherwise would have been indistinguishable using incubation time and PK-resistance as analysis criterion. However, this approach still failed to discriminate between closely related prions, although showing that PrP^Sc^ assemblies present different degree of stability in presence of chaotropic treatments that reveal epitopes [36].

Post translational protein modifications have been proposed to account for the PrP strain-to-strain variations. For instance, prion protein contains two glycosylation sites located in the structured C-terminal part of the protein. Both N-glycosylation sites are conserved in the *PrnP* gene among species, suggesting that N-glycans play an important role in the protein function. These sites, however, are not systematically glycosylated, as shown by the 3 detected bands in western blots performed with anti-PrP antibodies. Glycosylation pattern changes from one strain to the other, with variations in the relative abundance of the Di-, Mono- and Un-glycosylated forms and even the normal PrP^C^ glycosylation is differentially affected by depending on the prion strain [37]. However, glycosylation deficiency at either one or other glycosylation sites does not alter the susceptibility of the host to scrapie prions [38]. Moreover, strain characters were not modified when these glycosylation mutant mice were used for bio assays. This result was later supported by our work in vitro reporting that several PrP glycosylation mutants are faithful templates for PMCA (protein misfolding cyclic amplification, see Section 3.2.1) [39]. Other studies however reported that sialic acids that are deposited on glycan chains may significantly account in the prion replication: desialylated PrP^Sc^ was mainly found and eliminated in the liver while normal prions were targeted toward secondary lymphoid organs [40]. In the meantime, the desialylation of prions is reported to reduce the species barrier [41]. These data are particularly relevant with respect to cross-species transmission fate.

### 3.2. New Insights

#### 3.2.1. Templating Activity

In 1996, Prusiner’s team published the observation that FFI (Familial Fatal Insomnia) or sporadic CJD (sCJD) inoculation to a mouse expressing a chimeric human-mouse PrP gene reproduced both PK-resistant 19 kD deglycosylated band pattern for FFI and the 21 kD band pattern for the sCJD, respectively [42]. This basic observation paved the way to the concept of templating activity of prions. This concept has been explored in the field of yeast and fungi prions (the yeast prions will voluntarily not be documented here) but had been curiously neglected until the very recent years, when Baskakov’s team reported conformational switches within individual amyloids [43]. In this work, two strains of fibrils made from the identical recombinant hamster PrP showed various individual characteristics, derived only from the different conditions of formation: R strain was obtained under rotation of the monomers and displayed straight shape and polymorphous (twisted or not twisted morphology), while S strain was obtained under shaking and displayed a curvy simple line. When incubating these different fibril strains with heterologous mouse recombinant PrP monomers, they observed that whatever the original strain, the fibrils adopted the straight complex forms of the R phenotype. In addition, FTIR (Fourrier–transform Infra-Red) spectroscopy properties of the daughter fibrils were similar to the ones of the R phenotype. By contrast, R and S fibrils incubated with the homologous hamster monomers yielded the expected parental forms (microscopy and FTIR). Thus, monomer origin is able to imprint a new conformation and new properties to a given inoculum, resulting in the change or adaptation of the strain to its new host. However, these data also suggest that the host PrP could restrict rather than enlarge the conformational panel available for PrP^Sc^.

Owing to their very long incubation periods, prion diseases remain difficult to study in vivo. Although cell-based systems have been developed in several laboratories, they proved to be difficult to set up, particularly because this approach was not possible to implement to every kind of prion strain. Despite these difficulties, Weissman’s groups produced seminal data on prion strain adaptation and selection in vitro [44,45,46,47,48]. They first showed that neuron cell lines chronically infected by two prion strains (RML and 22 L) could be derived into several different cell lines with their own response to various other prion strains [44]. Although they could somehow stabilize cell-adapted prion features different from that of brain–adapted prions, they observed the occurrence of a “Darwinian selection” that allowed for the transition from one prion to the other [45]. Further selection could even be achieved using selective prion drugs inhibitors such as swainsonin, demonstrating that the strain features observed finely depend on the prion production conditions [45,48]. In such a context, Weissmann and colleagues further enforced the concept of quasi-species proposed by Collinge [49], consisting of a major component and many variants, which are constantly being generated and selected against in a particular environment: changing of conditions may result in the selection of a new variant with different features.

Several aspects of PMCA and RT-QuIC (real time quaking-induced conversion) amplifications have been studied and used for prion detection in body fluids or for assessing and validating decontamination procedures that will not be emphasized here. A focus on some of these topic has however been recently published [50]. PMCA approach was first described by C. Soto in 2001 [51] and involves the cyclic amplification of minute amounts of infectious material diluted in a brain lysate containing solubilized forms of PrP^C^. The repetition of a few seconds ultrasonication bursts followed by an incubation period at 37 °C produces the exponential transconformation of the PrP^C^ present in the normal brain lysate into a PK-resistant form. PrP^Sc^ is then detected on Dot or Western blot after PK-digestion. Practically, amplification factors and titration capabilities have been reached that extend far beyond that obtained with bioassays: 10^12^ amplifications were routinely obtained with laboratory scrapie strains [52] and the system allows for the amplification of a large number of strains, sometimes to a lesser extent, though. This reduced amplification level could however be largely compensated after several rounds of amplification. The difficulty to amplify sporadic MM1 CJD, appeared as a notable exception until Safar and colleagues reported that a modified PMCA using unglycosylated PrP was able to selectively amplify type 1 sCJD [53]. 

Castilla and colleagues proved the relatively high strain fidelity of the PMCA-driven prion amplification regarding currently available tools (incubation time, brain lesion scores, western blot profile, PK-resistance) [54,55]. Thus this in vitro amplification tool proved to be a valuable tool for the assessment of cross barrier crossing, a main advantage over bioassays being the extreme shortening of the time required for experimentation. Species barrier crossing could be demonstrated between mouse prion and “cervid” model mice, which would have required much more time with the bio assay [56]. 

PMCA has been described using brain lysate of transgenic mice as a source of healthy PrP^C^. But versatility of the system may be further increased by the use of cultured cell lysates. This was used in our lab to further dissect the requirements for PrP^Sc^ conversion [57,58]. Additional experiments determined that the conversion and amplification of recombinant PrP protein could occur in the presence of only RNA or phospholipids as adjuvant molecules [55,56,59,60,61,62].

Simultaneously, RT-QuIC was developed as another technique for the in vitro amplification of prion conversion [63]. In brief, recombinant PrP molecules are driven to fibrillation through alternative shaking and incubating with thioflavin-T (ThT) as a fluorescent marker: an increase in fluorescence emission, that could be observed in real time, is the sign of amyloid formation and PrP conversion. This approach proved to be sensitive enough to detect prion particles within blood cells [64] and diverse body fluids [65]. An important difference between PMCA and RT-QuIC is that the recombinant PrP that is converted in QuIC experiments is poorly infectious, whereas PMCA amplified products are usually as highly infectious as the inoculum. Noteworthy, this technique does not faithfully replicate the species barrier phenomenon that is recorded in target animals. Nonetheless, RT-QuIC has proved useful in discriminating among prion strains: Bank Vole PrP could be converted by almost every strain tested in RT-QuIC experiment. Lag phase and final fluorescence signals could be used for discrimination between different prion strains, though [13]. These observations parallel those made with transgenic mice expressing bank vole PrP [66]. On the contrary, the use of several different substrates could be used as a screen to differentiate between closely similar strains: atypical L-type BSE and classical BSE for instance [67].

A fluorescent approach to discriminate between prion strains was provided by a chemist group from Sweden using oligo-/poly-thiophene derivates [68]. Murine scrapie and CWD have been compared for excitation/emission spectra as well as fluorescence life-time of a few compounds: this parameter is modified in response to conformational restriction of the thiophene backbone following interaction with the different aggregates. All these methods could provide refined tools to differentiate strains that are difficult to by strain typing in animals.

#### 3.2.2. Size Distribution of Aggregates (Quaternary Structure)

**Figure 1:**
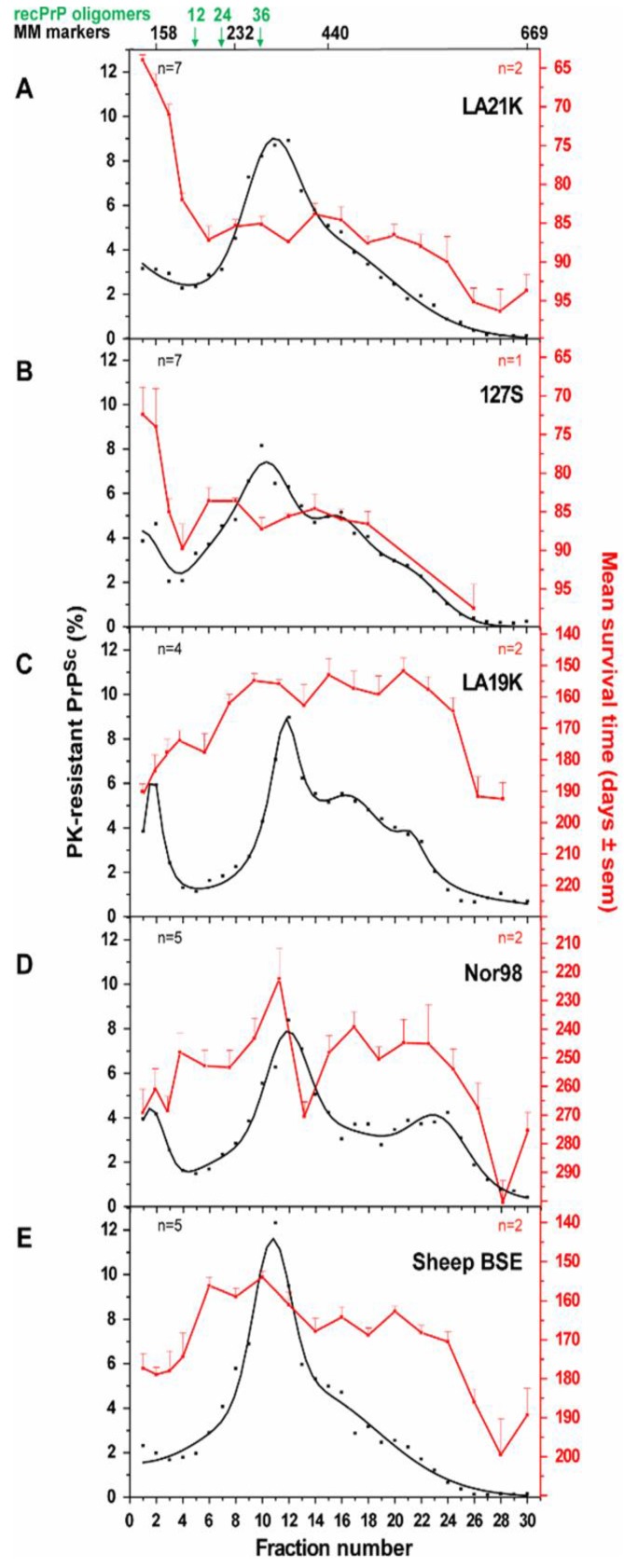
Brain homogenates from tg338 mice infected with LA21K (**A**); 127S (**B**); LA19K (**C**); Nor98 (**D**) and sheep BSE (**E**) were solubilized and fractionated by sedimentation velocity. Fractions collected from the gradient were analysed for PK-resistant PrP^Sc^ content (**black line**) and for infectivity (**red line**). For each fraction, the percentage of total PK-resistant PrP^Sc^ detected on the immunoblot is presented (**left axis**). For each fraction of each strain, infectivity was determined by measuring mean survival times in reporter tg338 mice (mean ± SEM; **right**, **red axis**). The sedimentation peaks of standard molecular mass markers (MM markers) are indicated on the top of the graph. From [71].

Biophysical approaches focused on size-distribution analysis of the prion particles. If not entirely carried on primary or secondary sequence, strain information should be somehow related to the tertiary or quaternary structure of the PrP^Sc^ assemblies. Several studies have already pointed the necessary role played by the PrP structure in the pathological process of prion transconformation [2,26,55,69,70]. Sedimentation velocity centrifugation in density gradients proved to be a valuable tool to separate and analyse prion fragments according to their size and/or shape, while preserving as much as possible the “natural” multimerization state of the prion particles and minimizing artefacts due to improper membrane solubilisation [71,72]: this later point is crucial for the reliability of the technique, since the presence of residual membrane lipids would modify the assemblies’ apparent density and lead to improper interpretation of the data. Sedimentation velocity-based fractionation will discriminate dense heavy aggregates that will sediment to the bottom of the gradient from the lighter fractions containing small aggregates or particles of low density. This technique allowed the precise discrimination between several ovine and hamster strains (Figure 1). The disconnection between infectivity level of the fractions (monitored by bioassay) and the PrP^Sc^ abundance (estimated by western blot) specifically for the ‘fast’ ovine and hamster strains constituted a striking finding of this approach [71,72]. These experiments led to the view that prions are formed of a strain-specified collection of non-uniform PrP^Sc^ assemblies with specific activities.

### 3.3. When Two Strains Look the Same

Box 1 **Of prion isolates, strains and types.**

***Of* *prion isolates, strains and types***

- Isolate: we refer here as to biological material that has been obtained through sampling of infected individuals;
- Strain: the term corresponds to a defined prion population isolated from one specified animal, with regards to the precision of the investigation technique: from basic observations (clinical signs incubation time and so on) to fine biochemical and biophysical parameters that are now becoming precise enough to allow for the discrimination of quasi-species within one strain; for the sake of simplicity, one regularly and erroneously omit the name of the host from which the strain has been originally isolated, even though a totally different prion population may have been selected when passed to the new host.
- Type: refers more particularly to a combination of biochemical parameters (mainly to the size of the unglycosylated PrP^Sc^ fragment after proteinase K partial digestion) that are independent from the host.

The prion phenotype characterization is as precise as the accuracy of observation tools used. In some cases, and despite the combination of several investigation methods, two strains may not be discriminated (box 1). For instance, it has been observed that some scrapie strains could display the same pattern than that of BSE . This raised the question whether BSE could in fact originate from a scrapie strain [73]. Later on, the identification of an atypical L-type BSE in bovine raised again doubts on the potential transmission of BSE to small ruminants [74]. First described within a flock in Italy, its behaviour in cattle is very different from that of the classical BSE (presence of amyloid plaques, low non-glycosylated PrP^Sc^ fragment). Upon passage on ovinized animals (VRQ allele), however, this strain turned out to be fully similar to C-BSE, which prompted the investigators to speculate that the mad cow outbreak of the late 80’s could have arisen from a passage by the small ruminants. In fact, these two strains, although they look virtually the same in the ovinized mice, keep their species characteristics, since they still can be differentiated when further back-passaged on bovinized mice (our unpublished observations). In addition, L-BSE could be differentiated from the classical one through its high propensity to colonize lymphoid compartments (our personal observations). Thus, study of prion replication in the lymphoid tissue and back-passage experiments (or to another intermediate species) can be useful to discriminate between truly identical strains and closely resembling strains.

## 4. To the Study of Species Barrier in Prion Transmission

What we currently know from the species barrier crossing is that the phenomenon can take a long time to be observed: adapted prions usually kill all the inoculated animals within a few weeks or months, out of a total incubation time that could range between 60 days for the fastest and up to the entire life of the animals (over 700 days for some mice); by contrast, non-adapted prions usually have incomplete attack rates and incubation times greatly increased and much more variable as compared to the original strain. During the adaptation process, however, these hallmarks faint and newly adapted prions recover full attack rate and reduced as well as highly reproducible incubation time, although several other factors may have been changed in the new host (final incubation time, tissue distribution, PK resistance…).

Strain-to-strain variation is often associated to the passing of one species to the other. In some instances, this can even result in the adaptation of several different strains: inoculation of transmissible mink encephalopathy (TME) to hamsters resulted in the selection of two different strains, depending on the dilution of the inoculum [75] (see Section 4.3 Coinfections). Recent work report on the influence of PrP^C^ expression level for the selection of different prion populations (see Section 4.4.1 PrP expression level) [76]. This is highly questioning in regards with the zoonotic and epidemic risks of such diseases (BSE, variant CJD, for instance).

### 4.1. Some Great Examples

Illustrating the complexity of the species barrier in frame with the prion strain characterization is a difficult task: a few examples are provided below, that have been chosen for the historical role they played in the prion field or because of their highly possible or demonstrated impact on human health (in terms of zoonotic risk).

Historically, one of the first prion species barrier to be studied with laboratory rodents has been the hamster to mouse transmission: early in the 70’s Kimberlin noticed that a hamster scrapie strain named 263 K hardly passed on mice [22,77]. However, years later, asymptomatic replication of hamster prions was demonstrated in mice [78,79]. Despite the absence of clinical signs, half of the inoculated mice had detectable levels of PrP^Sc^ in the brain and also showed the presence of specific prion disease lesions in the brain. This work was the first to further include the search for prion specific signature in the target organ with or without clinical signs for the evaluation of the barrier species. In seminal experiments, the use of transgenic mice expressing hamster PrP allowed hamster prion transmission to mice, indicating that PrP amino acid differences contributed to the species barrier [80]. Then, PMCA approaches confirmed that mouse (RML strain) prions could progressively be adapted to hamster species [81]. The in vitro adaptation could be achieved within 4–6 rounds (~2 weeks, while in vivo adaptation would have required more than 3 years). Similarly, hamster prions (263 K strain) could be adapted to mouse. Interestingly, both directions of adaptation yielded prions showing fine variations in incubation time/PK-resistance/brain deposition pattern/glycosylation profile, etc. These results suggest that although very similar to what is observed in vivo, PMCA-driven adaptation process is different from what is observed in living conditions. 

Mad cow disease in the 90s triggered very intense research on the mechanisms involved in the crossing of the species barrier for some prion strains. Investigating the capacity of BSE prions to propagate in new host species led to the initial conclusion that the BSE agent was not able to infect mice expressing only human PrP (HuPrP^+/+^ PrnP^0/0^, valine allele at position 129) [1]. Looking closer, however, revealed that the BSE prions finally needed more time than CJD prions to install and replicate in mice expressing human PrP. The same lab detected transmission of BSE to macaque or cats, while their PrnP^0/0^ HuPrP^+/+^ still were alive (after more than 500 days post-inoculation) [2]. Thus, BSE prions were more and more amenable to infect humanized animals. The year after, Bruce and colleagues reported that both BSE and vCJD agents replicated similarly in certain lines of conventional mice and induced the same strain phenotype, thus strengthening the link between the epizootic burst of BSE and the outbreak of British and French vCJD cases [82]. A few years later, Prusiner’s team provided another evidence that BSE and vCJD were etiologically linked [83]: they observed that bovinized mice developed similar pathologies when inoculated with either BSE or vCJD agents (incubation time, lesion distribution and score, PK-resistance profile), these profiles being absolutely different from those observed after scrapie infection. These observations confirm that two agents isolated from different species could eventually be the same strain. Other lines of transgenic mice overexpressing the Met allele of human PrP were developed and further provide evidence of the intricate link between BSE and vCJD. In two lines, the disease occurred at incomplete penetrance and was mostly subclinical [84,85]. However, one of the lines showed a higher attack rate extraneurally in the spleen tissue (see below). 

The cross-species capacities and zoonotic potential of CWD prions are another emerging public health concern. This disease affecting cervids is known for long but got only recently media coverage when concerns about the passage from wild ruminants to human started to get conceivable. While CWD was reported in the early 90’s to mainly circulate between captive wild cervids, [86] a warning was raised against the possible epidemic extension of the disease, which truly occurred in the USA during the last 3 decades (reviewed by Watts [87]) and even recently popped up in Scandinavia [88]. Considering the data obtained with scrapie and particularly with the fact that some prion strains apparently can circulate without species barrier, it was of importance to determine to what extent this CWD agent is confined to wild cervids. It is already known that CWD can adapt to certain strains of mice, expressing high level of murine PrP [89]. After one passage, strain seems to be stabilized, with mice repeatedly succumbing 220 days after inoculation, all other features of the disease were looking similar to the genuine CWD, including the spleen tropism. Recent data further evidenced a threat of possible transmission to humans: Herbst and colleagues described the different behaviours of two different CWD strains—CDW1 strain could pass to hamsters but not to mice. By contrast H95+ strain could infect efficiently the mice, while the hamster was less susceptible [90]. Humans’ natural resistance to CWD infection has been hypothesized to rely on a specific amino acid stretch in the β2 loop of the PrP; by swapping this domain with that of elk PrP, Kurt and colleagues rendered the mice expressing this mutant PrP fully susceptible to CWD [91]. Notably, susceptibility to CJD was inversely reduced. A very recent work presented to the Neuroprion meeting reports that CWD can be passed orally to cynomologus macaques [92].

Conversely, there are host species that are susceptible to almost every strain: for example, bank vole rodent turned out to stand as a mammal particularly tolerant to many prions [93], including notably sporadic CJD [11]. Transmission of these prions to bank voles results in a disease at full attack rate, with little or no species barrier and in a tempo similar to transgenic mice overexpressing human PrP. Bank vole and human PrP amino acid sequence are differing by 12%. This indicates that the bank vole PrP conformation is *per se* prone to conversion by human CJD prions. Recent transmission of a library of prion strains to transgenic mice expressing bank vole PrP (M109) further support the view that bank vole PrP can be converted by many abnormal PrP^Sc^ conformations. To strengthen the demonstration, so-called ‘resistant’ animals could be rendered compliant with prion infection after introgression of a genuine or modified prion protein—for instance, the Drowsy strain affecting Syrian hamsters but not Wild-Type (WT) mice could readily infect mice expressing a chimeric hamster-mouse PrP [94].

The species barriers depend in part on host’s PrP primary structure and in part on the strain itself. Up to date, every one of these species barriers has shown they could be crossed, provided that enough time, correct animal or organ target and passage numbers have been taken into account: hamster prions can pass onto mice [95]; TME can pass to cattle [14]; BSE and vCJD both can adapt to Guinea Pig [96]; CWD transmission from cervids to human is every day more probable, at least under certain specified conditions [97]: CWD can pass in vitro with PMCA to human brain homogenate but efficiency is greatly enhanced if amplification/adaptation of the CWD prion has been previously performed through PMCA using CWD template [98]; experimental infections to non-primate monkeys were also efficient [99,100]. In conclusion, the strength of the species barrier is mainly dependent on the parameters that are addressed: a closer look to subclinical disease either centrally or peripherally may lead to the assumption that there is finally no absolute species barrier, at least to the experimental level. 

What are the factors that render prions adaptable to their new host? What features allow BSE to stand today as the sole prion that adapts easily to other species? Despite significant efforts to improve our knowledge, the answer is still largely unknown. However, several leads have been followed that may help building models for the structure of what we call PrP^Sc^. Some of these models will be evoked at the end of the paper but the few following sections will first focus on the experimental parameters that demonstrated their influence on the strength of the barrier species.

### 4.2. Importance of Primary Sequence, Aminoacid Polymorphism

The primary sequence of the PrP stands naturally in front line, since it is accepted by almost all the scientific community that this endogenous protein is the only responsible for the disease. The primary sequence (allelic variations, point mutations) would obviously be the main vector ruling the host’s susceptibility to a given strain. With the introduction of transgenic animals, it became easy to test whether one given PrP sequence could account for the susceptibility to prion diseases: several studies report the acquired susceptibility of mice following expression of recombinant or chimeric hamster protein [101,102]. Homology between the inoculated prion and the host’s PrP looks like a prerequisite [80,103,104]. In the latter case, transgenic rabbits expressing ovine PrP were fully susceptible to scrapie, showing that the rabbit environment is not, *per se*, incompatible with prion transconformation, although the animal is known to be naturally resistant to prion infection. However, with the notable example of the bank vole being a universal acceptor, the common view is that structural compatibility between host PrP and the infecting prion strain governs the cross-species transmission of prions.

Polymorphism within one species have a dramatic impact on the susceptibility of the host (see for instance how ovine polymorphism is governing scrapie transmission [105,106]). The extreme cases are illustrated by the spontaneous prion conversion attributable to familial point mutation of the human PrP gene responsible for genetic CJD or FFI (for a review, see [107]), or by the I109 M point mutation affecting the bank vole PrP: mice expressing I109 Bank vole PrP spontaneously develop a prion disease within 4 months of age [108]. This phenomenon does not strictly apply to the species barrier paradigm. However, it had been shown that the mutations reported in humans seem to cluster in several groups depending on their ability to cross species barrier and infect mice [109]. In that particular case, species barrier was regarded as a tool for the characterization of different prions strains. From an epidemiological and clinical point of view, it is relevant to consider these point mutations, since they condition full sensitivity or resistance to prion disease. The homozygous methionine or valine in position 129 of the human PrP is determinant for the sensitivity to CJD [110]. To date, all clinical cases of vCJD have only occurred in patients homozygous for methionine at codon 129 [111]. However the V129 genotype does not protect against vCJD, despite full protection against BSE [112] Polymorphism in position 219 (E/K) is also associated with resistance to prion infection in Asian population [113]. More recently a G/V polymorphism has also been reported to be responsible for full resistance to Kuru infection and was proposed to result from naturally-driven selection process that increased resistance against Kuru in the exposed population [114].

In addition to governing intra species susceptibility to prion diseases PrP polymorphism greatly influences the susceptibility of the host to exogenous prions. Noteworthy however, this polymorphism has an impact on the population genetics, with breeds being historically more susceptible to scrapie than others because of genotype variations in positions 136/154/171. The M/V polymorphism at position 129 for human PrP similarly controls the susceptibility of individuals to vCJD [110]. Green et al. report that the elk prion codon 132 polymorphism controls cervid and scrapie prion propagation [115]. In brief, transgenic mice expressing the elk mutant L132 are resistant to CWD prion inoculation (no clinical sign at up to 600 days post infection). However, the authors report the detection of small amounts of PK-resistant PrP^Sc^ in the brains of the animals inoculated with M/M132 inoculum but not with M/L132. This suggests that L132 genotype is not resistant to infection but rather requires adaptation passages in order to select a fully adapted prion. It is worth noting that in contrast to elk prions, hamster prions adapted easily to the 132 L elk PrP. Using the Bank vole as a model, Agrimi’s team observed that I/M109 heterozygote animals incorporated equal quantities of both allelic forms of PrP in the prion particles [116]. Thus, at least in some cases, it seems that polymorphism does not entirely rule the susceptibility to prions.

Beside natural polymorphism, experiments have been performed to test for the mutual influence of different PrP molecules to diverse strains of prions. Several experiments of cross species inoculation have been performed by Kimberlin and colleagues to assess the infectious potential of scrapie prions to infect mice, hamsters, etc. [117,118,119]. Later, when the first transgenic mice started to be available, the transgenic PrP was co expressed along with the endogenous one. It rapidly appeared that the expression of both the endogenous and the transgene could result in odd responses to prion infection. Chesebro’s team published several reports mentioning the interference of heterologous PrP on the accumulation of prions in a cell culture assay [120,121]. A single amino acid substitution could be responsible for drastic inhibition of prion production in cell culture. But other studies also report on the influence of additional factors for the efficient replication of prions [122]. These observations paved the way for a long series of publications mentioning a hypothetical protein X as cofactor in the prion mechanism [123]. But recent in vitro conversion experiments invalidated this hypothesis [124].

### 4.3. Co-Infections

The influence of co-infection, although not strictly relevant in the species barrier topic, is questioning: since several prion strains can infect a given host, what could a multiple infection look like? One would logically suppose for example that the fastest strain would take over the slowest strain. Surprisingly it happened to be quite the opposite: fast strain 22A inoculated to mice that had first received a slow 22C strain showed delayed incubation periods [125]; when a Hyper/Drowsy combination of TME prions was peripherally inoculated to hamster, the fastest Hyper strain progression was delayed when co-administered with the slowest strain Drowsy [126]. The authors proposed a competition between both strains for a limiting PrP resource [127].This competition did not occur when both prions were simultaneously inoculated at different locations [128].

### 4.4. Influence of Expression Level and Post Translational Modifications

Considering there is a competition for substrate between two different strains, then the amount of available PrP certainly influences the susceptibility, or to a lesser extent the kinetic of the disease. In addition, the several post translational modifications that undergoes the PrP^C^ during its journey toward the membrane may also influence the susceptibility/convertibility of PrP to prions. 

#### 4.4.1. PrP Expression Level

The influence of PrP^C^ expression level has been assessed on incubation time, infectivity titre and lesion distribution in 3 strains of mice, whose expression level varied from ½ to 8x [129]. The incubation period was reduced when PrP^C^ level was increased: during an exponential phase, rapid accumulation of PK-resistant PrP^Sc^ occurs, followed by a plateau phase, whose length is proportional to the PrP^C^ expression level. The authors suggest the accumulation of toxic forms of the PrP that ultimately induce the clinical signs once a certain threshold has been reached. Recently, our group reported the characterization of three independent prions selected from a single scrapie isolate inoculated to transgenic mice expressing various levels of strictly homologous VRQ allelic forms of ovine PrP. The strains were mainly phenotyped according to their PK-digestion profile and their incubation duration [76]. Upon transmission of a 21 K isolate to overexpressing mice (>3.5×), a new 19 K phenotype is progressively selected, with frequency and PrP levels raising accordingly. A third phenotype (21 K fast) is reported to emerge in a stochastic fashion that outcompetes 19 K prions in high expressor hosts.

Although these studies were done in homotypic transmission context, they highlight the key role of PrP^C^ levels in the disease tempo and in the prion selection and emergence. It is likely that PrP^C^ would similarly be at play in heterotypic transmission events.

#### 4.4.2. Secondary Modifications: Glycosylation, Sialylation, Protease Digestion…

Glycosylations and other post translational processings have been shown to play a significant role in the transmission and adaptation of prions to a new host. Recent work with PrP mutated to the first or the second glycosylation site dramatically increased or suppressed the species barrier upon infection with 2 human prions (MM2CJD and vCJD) or 263 K hamster scrapie [130]. It is worth noting, however, that the amino acid mutations designed for glycosylation alterations may also account for the observed phenotype [131]. In vitro conversion experiments using hamster prions in presence of various mouse PrP constructs suggest that heterologous conversion favours unglycosylated PrP incorporation, while autologous conversion results in the usual 3-banded profile [132]. Glycosylations may be further modified through sialylation of the sugars. It has recently been shown that sialylation of prion protein could modify the species barrier [41]: while normal mouse brain homogenate needed more than 4 rounds of PMCA to reach a steady state level of amplification using a 263 K hamster strain, the same desialylated brain homogenate reaches the plateau in one single round. Reciprocally, hamster brain homogenate failed to amplify 22L or ME7 mouse strains even after 10 rounds but upon desialylation, the PMCA reaches the plateau in 3–4 rounds. It is furthermore shown that sialylation participates in a host/tissue and cell-specific manner to the regulation of PrP [40,133]. Sialylation process is shown to occur even after the PrP has been converted [40,134].

GlycoPhosphatidyl Inositol (GPI) anchor, which attaches PrP to the membrane through its C-terminus participates also to the conformational landscape of the prions upon infection: upon passage on GPI^-/-^ mice, most of the prions retained their specific characteristics when passaged back to their original host, except CWD which gain in PK resistance and chaotropic [GdnHCl]_1/2_.stability [135].

### 4.5. Prion Route May Influence Prion Transmission Fate

Lymphoid tropism of some prion strains has been described for a long time [24]. Prions have been shown to replicate and accumulate in follicular dendritic cells (FDC) from the germinal centres of lymph nodes and the spleen. Several other immune cells (like B-cells, macrophages) have been shown to carry prion infectivity, however, it has been demonstrated that FDCs are necessary and sufficient for prion replication in the spleen [136]. Of note, the FDCs are not of lymphoid nor myeloid origin, despite their pivotal role in the initiation and maintenance of immune response: rather, they derive from sub-endothelial cells and differentiate upon trophic interactions with B-cells [137]. Macrophages and B-cells also stain positive for PrP^Sc^ but they are mainly involved in PrP^Sc^ scavenging and transport, respectively [138,139]. Therefore, lymphoid compartment will have a balanced influence on peripherally acquired prion fate: in one hand, macrophage and in particular splenic scavenging functions will actively degrade or neutralize prion infectivity, while, on the other hand, FDCs will actively replicate prions that are then shuttled to the terminal nerves ending near the germinal centres [140]. Thus, the resulting lymphoid tropism could be the net result from this balance, as claimed recently by Bartz and colleagues [141], who were able to detect Drowsy infectivity within hours after inoculation but also reported that these prions disappeared thereafter as a consequence of an increased susceptibility to proteases (in vitro PK assay). Prion replication in periphery could otherwise be dictated by strain features. It is for instance well known that vCJD, as opposed to other strains of CJD remarkably replicate within the human lymphoid system [142]. Thus, oral contamination by the vCJD strain, which targets the Peyer’s patches beneath the jejunal epithelium, is much more prone to occur than with other sporadic forms of CJD. However, recent analysis of the last documented Kuru case (in 2003) reveals that despite a certain food borne contamination, the Kuru patient did not exhibit the marked vCJD-typical colonization of the digestive tract. This suggests that peripheral pathogenesis of Kuru is similar to that seen in classical CJD rather than vCJD [143], although a more recent study finally considered that sCJD and vCJD accumulated similarly in the lymphoid system [144]. Thus, strain lymphotropism does not necessarily reflect the preferred inoculation route. This question had been addressed with comparison of oral and intracerebral routes of BSE inoculation in macaque as a model of species barrier transmission [145,146] and the general conclusions that may be derived from these studies is that lymphoid tropism does not facilitate, *per se*, the crossing of barrier species.

### 4.6. Immune Status, Age of the Host

It is for long known that immune status and host age or developmental stage influence the PrP expression in target organs such as the brain but also and particularly the lymphoid organs [147,148,149]. As a consequence, susceptibility of young sheep to oral contamination with BSE is drastically decreased after weaning [150]. On the other side of the lifespan, aged mice infected intraperitoneally with RML prions show significant longer incubation time than their younger littermates [151]. This is to be related to the decrease of follicular germinal centres with age [152]. Thus, depending on the age at the time of infection, prion replication may be significantly affected, with obvious effects on the crossing (or not) of the species barrier. For instance, the transmission of BSE was much more efficient on young mice, while older animals remained free of clinical sign all along their lifespan [153]. Noteworthy, some aged mice could still replicate at visible levels the prion in their spleen. Thus, in that particular case, the modification of the species barrier should be regarded as a consequence of a receptor abundance modulation. Still, these observations are of interest when considering the exposed groups within the whole population.

### 4.7. Cell/organ Selectivity

**Figure 2:**
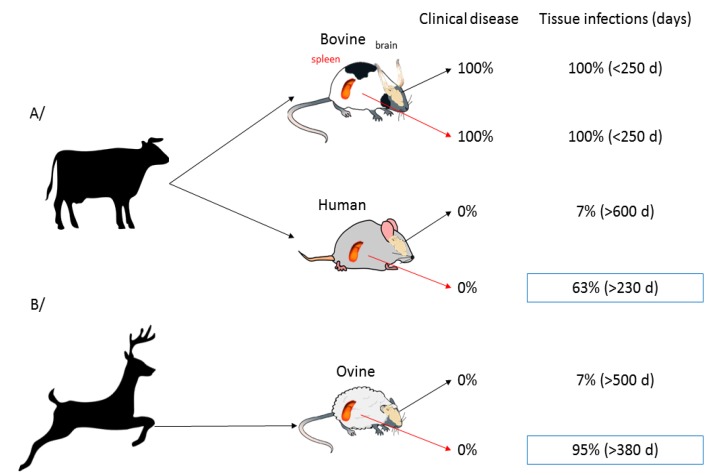
The spleen is much more permissive than the brain to the passage of heterologous prions: percentage of diseased animals and western Blot-positive tissue of BSE (**A**) or CWD (**B**) prion agents inoculated intraperitoneally to bovinized, humanized or ovinized mice. Spleen and brain were collected at the death of the animals. Tissue infection was diagnosed upon the detection of PrP^Sc^ by western blot. From [85].

As evoked in the previous section, prions can replicate and their expression could be drastically modulated in several organs outside the central nervous system. In addition to that, peripheral organs may, *per se*, have a different behaviour with respect to the invading prion and the inoculation route. These observations have been made in our laboratory when we monitored the fate of a scrapie prion strain following inoculation by intracranial or intraperitoneal routes [154]: the disease greatly differed in clinical signs, abnormal prion protein levels and neuropathology. In another study, we monitored brain and spleen of ovinized and humanized mice for the presence of infectivity or PK resistant PrP after inoculation with hamster sc237, CWD or BSE [85]. Overall, the three strains, which are not transmissible to either ovine or human PrP mice, did not indeed replicate efficiently in the brains of the inoculated animals: for instance, only 2 out of 29 ovine mice infected with CWD were positive by western blot at the end of their lifespan. By contrast, spleens of these animals were almost consistently positive (Figure 2) and from the early time of infection onward. Overall, the spleen appeared 9–10 fold more permissive than the brain to foreign prions. The reason for such tissue-dependent strength of the species barrier remains to be determined. Spleen PrP^C^ might be more prone to heterotypic conversion than brain PrP^C^, due to conformational variations. The spleen environment might constitute a better niche, due to prolonged possibility of interactions between brain and spleen (through axonal terminations located close to the germinal centres) or presence of co-factors, such as complement. Ultimately, absence of cell prion toxicity outside the brain could also account for an efficient replication of PrP^Sc^. Whatever the reasons, these features allow prion extending its host range. It also provides and experimental explanation to the high number of asymptomatic individuals exposed to BSE agent in the UK population showing pronounced accumulation of PrP^Sc^ in their lymphoid tissue [155]. 

Other hypotheses involve a tissue specific clearance metabolism as responsible for the different strain tropism [141]. As mentioned previously in a former section, the authors describe the successful PMCA amplification of Drowsy TME in the spleen of peripherally-inoculated hamsters, although this strain was not supposed to replicate in that tissue. They conclude that the strain selectivity against Drowsy prions in the lymphoid organs is a consequence of the strain-specific efficient removal of the infectious material. This hypothesis however does not seem to be valid when brain prion distribution was concerned [156,157]: both studies argue at 20 years interval and with different tools against a brain tissue selectivity. Cell culture experiments have also been set up in order to evaluate strain selectivity but the overall resistance of cell lines or even primary cultures to prion infection remains to be overridden [158].

## 5. Consequences on Prion Adaptation to New Host

Following a substantial species barrier, different scenarios may be possible: **(i) Silent passing**—The prion may be silently passing as it does when Sc237 is inoculated to ovine PrP mice (see previous section). Several studies have reported that some strains require very long adaptation periods and iterative passaging in order to successfully replicate in one given host [159,160,161]. Three or more reinoculation steps are often needed to get successful isolation of poor prion transmitters. In such cases, the receptor animals also play a pivotal role, in particular with respect to the amount of target PrP^C^ that could be expressed. In some instances, the resulting prions retain the features of the parental strain [159]. In other instances, the isolated prions differ significantly from the source inoculum [161]. **(ii) Progressive evolution—Mutational events**. In that case, evolution could concern more than two species: for instance, when questioning the origin of the BSE and vCJD epidemics that hit Western Europe a few decades ago. An atypical form of BSE (BASE) has been shown to evolve into a form that is indistinguishable from BSE in wild-type mice [162]. Others described the progressive evolution from a H-BSE to a classical BSE when the strain was serially inoculated to mice [163]. It has also been reported that passage through an intermediate small ruminant allows BSE to better adapt to humanized mice [164,165]. Overall, BSE prions, whether atypical or classical, seem to display a unique behaviour with regards to species barrier, since it is the only prion strain capable of adaptation to a great variety of hosts [166]. In other situations, evolution may be more abrupt (incubation duration deceased rapidly between 1st and 2nd passage: see for instance, the emergence of T1-Ov and T2-Ov strains following experimental passage of MM2 CJD to ovine PrP mice) [167]. The progressive evolution of prions was further supported by Weissmann’s group, who argued for a Darwinian evolution of prions, through silent (or not) “mutations” [45]. These data were supported by in vitro experiments that establish prion evolution induced by chemical prionostatic drugs and selection of prion ‘quasi species’ from an initially homogenous prion strain [168]. As mentioned earlier, other groups support the concept of a ‘portfolio of conformations’ for a given strain, that could match (or not) with a portfolio of possible conformations for the receptor PrP [49,169]. The observation that H-BSE phenotype is lost upon passage to hamster mice and restored when passaged back to bovine fits this model [170]. An evolution of the concept emerged from the observation that two strains could too closely look similar to be distinguished, albeit showing different incubation times. In addition, these strains could constantly evolve form one strain to the other, rendering them impossible to clone [171]. 

### 5.1. De Novo Synthesis and In Vitro Assessment of Species Barrier

The experiments reported in the previous sections required the massive use of laboratory animals for the evaluation of species barrier. However, even with the use of transgenic mice that considerably shorten the incubation time in comparison to what is recorded with the target animals, the time needed to obtain such data with 4 and even 5 successive passages is extremely long, not to mention the high number of animals to be included… For that reason, alternative in vitro methods were eagerly needed. Most of them were conducted using PMCA or QuIC methods. As a recent and comprehensive review has recently been published on the topic [172], we will mainly focus on the latest highlights of in vitro transmission barrier studies. 

The ability of PMCA to create de novo prions was certainly one of the most interesting contribution of this technique [62]. Besides the definitive proof of the proteinaceous origin of the prion disease, it allowed to determine a few RNA and phospholipid cofactors that are crucially needed for the conversion to take place. But several other cofactors have been evaluated, whose influence in strain selection appeared to be determinant [173,174]. In that later case, removal of phosphatidylserine provoked the phenotypic convergence of the 3 strains tested, as well as a 5 log_10_ reduction in infectivity. This phenotypic convergence has been reported elsewhere [175], suggesting that the in vitro environment needs to be better controlled in order to properly mimic what is observed in vivo. In addition to these fidelity problems, several studies mentioned the reduction or absence of infectivity resulting from the QuIC amplification of prions [176,177].

### 5.2. In Vitro Assessment of High Species Barrier

Barria et al. reported the generation of cervid prions that can replicate on human matrix, provided that they have been previously submitted to 2 rounds of PMCA using cervid brain lysate [98]. Conversely, PMCA amplification of 263K scrapie using partially deglycosylated PrP matrix produced a mixture of classical and atypical PrP^Sc^ profiles that suggested the development of a new strain. If true, however, this new strain was fully restored to the classical 263 K profile after a series of 10 rounds in classical conditions [178]. PMCA was also used to generate in vitro prions using PrP from mammals known to be naturally resistant to the disease [179,180,181]. In addition to prove that allegedly resistant animals produced transconformation-prone PrP proteins, these studies established that BSE could induce in vitro the conversion of these so-called resistant PrP and that this PrP^Sc^ was fully infectious when administered to the target animal. Similarly, PMCA was used to render mice susceptible to hamster prions and vice versa [95]. While attempting to reduce the number of passages needed for the adaptation of scrapie strains to ovine mice, it was noted that some strains were compliant with a PMCA shortcut, while others produced prions with divergent features [182]. PMCA should therefore not be regarded as a fully effective method to faithfully reproduce the prions obtains with bioassays. 

## 6. Proposed Mechanisms for PrP Conversion, Strain Determination and Species Barrier Crossing

Despite this significant amount of experimental data, we still are waiting for some unifying model for prion conversion. Several hypotheses have been proposed for the transconformation and the elongation of infectious prions following the introduction of a seed. A first generation of models, based on a Prusiner’s hypothesis [183] invokes the faithful reproduction of a template. This model however does not account for the emergence of variant prions strains from a given cloned parent. Therefore, a mechanism should be at work to allow this diversity to occur. 

### 6.1. Prion Diversity from a Structural Portfolio and Selection of Mutants upon Species Barrier Crossing

Collinge and Clarke proposed in 2007 a model where prions are described as a panel of thermodynamically favourable conformations, referred to as a portfolio, of which some structures may or may not be selected when passing from one host to another [49]. According to this hypothesis, the strength of the transmission barrier reflects the overlap between the available portfolios of a given primary PrP sequence in two different hosts: the larger they overlap, the lower the species barrier. Some questions still remain to be addressed: in particular, what were the mechanisms at work in the generation of this primary diversity and why the resulting phenotype always displays clonal properties. In addition, observations with promiscuous strains like BSE or universal acceptors like the Bank vole question this view: for instance, one should expect from the bank vole that accepts almost every prion to display a conformational portfolio that includes most of the prion strains portfolios; if true, then no change should be seen when the strains will be passed from an host to the bank vole, which eventually was not the case [12]. When passed on bank vole, most of the strains showed an evolution in their characters but each time the character that emerged was alone, as if every other conformational state was drastically silenced (although it could express in the context of another inoculated prion). Therefore, we still needed a model that fits the puzzling data. The following sections will delineate the factors that have been taken into account in the available studies. 

### 6.2. Deformed Templating

**Figure 3:**
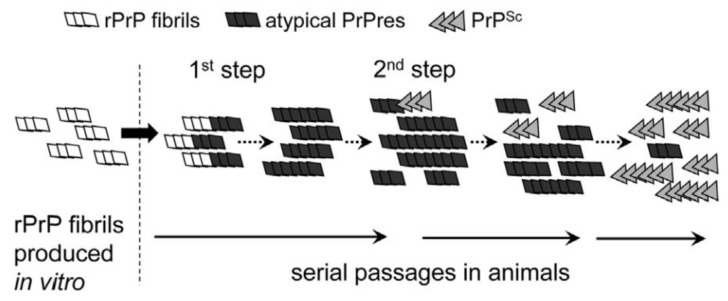
Schematic presentation of the mechanism, illustrating genesis of PrP^Sc^ triggered by rPrP fibrils. In a first step, rPrP fibrils seeded atypical PrP^res^, a transmissible form of PrP that replicates silently without causing clinical disease. Replication of atypical PrP^res^ occasionally produces PrP^Sc^ in seeding events that appears to be rare and stochastic as described for a deformed templating mechanism. PrP^Sc^ replicates faster than atypical PrP^res^ and eventually replaces it during serial passages. The two forms atypical PrP^res^ and PrP^Sc^ can be distinguished after PK treatment via staining Western blot analyses with discriminating antibodies. Atypical PrP^res^, alternative self-replicating state of prion protein; PrP^Sc^, prion protein scrapie isoform; rPrP, recombinant prion protein. From [185].

Based on observations published in 2012, Makarava and colleagues proposed a model in which recombinant hamster fibrils induce PrP^C^ to form what they call an atypical PrP^res^ [184]. In that model, two major successive steps are at work for the induction of PrP^Sc^ and the generation of a new strain of prion: in their experiment, the first step results from the formation of atypical PrP^res^ triggered by 0.5 M GdnHCl. This atypical PrP^res^ was detected in the brain of mice at their end of life but was not associated to any clinical sign. Then, the PrP^res^ could trigger the slow conversion of PrP^C^ into PrP^Sc^. The kinetics for the production of both PrP^res^ and PrP^Sc^ are quite different: generation of atypical PrP^res^ is RNA independent and its FTIR structure closely resembles that of parental fibrils; the second step, which was named “deformed templating” is more stochastic and less described. It is postulated that the strain structural diversity is acquired depending on the environmental constraints. The authors added that once formed, PrP^Sc^ does not require atypical PrP^res^ anymore and outcompetes its rival thanks to favourable kinetic constants (Figure 3). Since these two steps are independent, they could occur in separate animals. This hypothesis would thus explain why the crossing of the species barrier may be achieved even after several successive passages in recipient animals in which no classical PrP^Sc^ could be detected. According to Makarava, though, one should be able to detect the atypical PrP^res^. At the moment, this was confirmed in the case of 263 K scrapie prions [185]. In addition, the authors propose that, in contrast to the first step, the rate of deformed templating is not influenced by PrP^C^ concentration. This model does not address however the mechanisms that concur to the production of this atypical PrP^res^.

In an attempt to obtain insight into the quaternary structure of the PrP^Sc^ assemblies, our team recently published data obtained using velocity sedimentation gradients on urea-denatured and refolded purified PrP^Sc^ associated to the assessment of their specific infectivity [186]: we demonstrated the existence of stable packs of oligomeric subunits (suPrP) that encode the main strain structural determinants: when PrP^Sc^ aggregates were denatured under increasing concentrations of urea, the velocity sedimentation gradients evolved from large polydisperse aggregates toward the generation of small elements, presumably trimers that were named suPrP. Upon dialysis refolding, the velocity sedimentation gradients identified condensation of refolded aggregates (rfPrP) but with a different distribution from that before denaturation. SuPrP bricks turned out to be fully PK-sensitive and unable to template infectivity either in vitro or in bio assays. However, upon condensation the suPrP bricks regained full infectivity and PK-resistance properties of the parental strain. One of the most important findings was the fact that suPrP, rfPrP and PrP^Sc^ shared a dynamic equilibrium: upon dilution of 263 K PrP^Sc^ in physiological buffer, a rapid decrease of the light scattered by the oligomer solutions showed a significant reduction in the size of the particles, resulting from the dissociation of the PrP^Sc^ into suPrP. When local suPrP concentration was restored and urea removed, condensation of the suPrP into rfPrP could be observed by western blot and infectivity restored as assessed with PMCA. 

**Figure 4:**
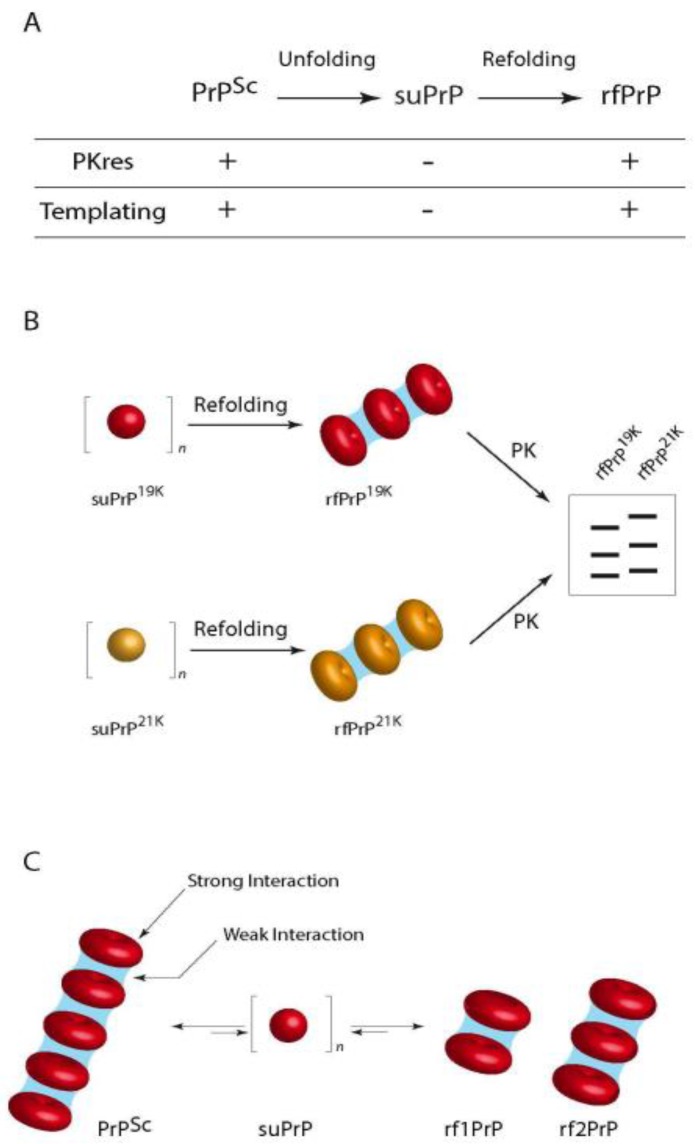
The role of suPrP in the dynamics of PrP^Sc^ assemblies. (**A**) Evolution of PK resistance and templating propensities of different types of PrP assemblies obtained after sequential unfolding and refolding of the parental prion. PrP^Sc^ is the native prion; suPrP is the elementary oligomeric PrP subunit; and rfPrP is the refolded conformer formed after the polymerization of suPrP. The process of conversion of suPrP into rfPrP requires a conformational change in the PrP protomer of suPrP (represented here as a sphere) to form infectious and PK-resistant assemblies (represented as stack of torus); (**B**) The conserved differential proteolytic pattern of rfPrPT1-Ov-21K and rfPrPT2-Ov-19K suggests that their respective suPrPs (represented respectively as yellow and red spheres) exhibit distinct conformations. During the refolding step (**C**), two modes of organization contribute to the cohesion within PrP^Sc^ assemblies. Weak interactions (in blue) are involved in maintaining the overall quaternary structure by stacking suPrPs, when strong interactions are involved in the cohesion of PrP protomers in suPrP oligomers. The weakness of the interactions interlinking suPrP means that PrP^Sc^ assembly and disassembly are highly dynamic events, even in the absence of a chaotropic agent and free suPrP could exist in equilibrium with infectious assemblies. From [186].

Thus, the results presented in this work suggest the existence of two organization levels within prion assemblies (Figure 4), one suPrP oligomeric subunit (that could contain 3–5 monomers) and a meta assembly that gathers the suPrP subunits and supports the strain infectivity level and structural conformation features. Whether the suPrP pre-exist to the PrP^Sc^ before being included in the elongating polymer or the suPrP results from the incorporation of PrP^C^ into the polymer remains to be addressed. The former hypothesis however implies that PrP^C^ and suPrP shall be separated in normal conditions.

This mechanistic proposition for the generation of elementary infectious prion bricks that co-exist as an equilibrium with larger assemblies is compatible with the portfolio model of Collinge. We propose the initial coexistence of several structurally different prions within a single brain homogenate [76]: the emergence of a new strain after prion inoculation to a strictly homologous recipient animal results from a difference in PrP^C^ expression level between inoculum donor and the recipient transgenic mice; the original 21 K strain may be favoured in low PrP expressor animals because the elementary brick could be more efficient at recruiting PrP^C^ at low concentration, while the bricks that lead to 19 K phenotype would benefit the advantage of high PrP expression. Alternatively, stochastic events mays also produce a third strain that could outcompete the two others.

It is probable that in a heterologous transmission, prion inoculum will first depolymerize just after injection and produce the main suPrP that is observed in the gradient experiments. Then, the elementary bricks would have to recruit PrP^C^ that may or may not accommodate the suPrP. This phenomenon could be highly stochastic, the probability that host PrP adopts a conformational state compatible with the foreign suPrP should be related to the proximity of prion strain and host. The generated assemblies could be rapidly stabilized and amplified, thus producing an infection with no apparent species barrier; conversely, when host PrP could not fit the topological constraints imposed by inoculum suPrP, the process would need longer time to produce and test pseudo stabilized oligomers or new suPrP that would ultimately emerge as a new prion strain.

## 7. Conclusions

Prion strains and species barrier phenomena still remain difficult questions to address. Much knowledge has been gained regarding the characterization of the strains (thanks in particular to the PMCA in vitro methods and to the gradient fractionation techniques). The model proposed by our lab identified some features for the faithful reproduction of prions and proposed a kind of generic polymerization principle where prion fibres exist in equilibrium with one or possibly several sub-units that contain the structural strain determinants. Crossing of species barrier could result from the emergence of one of these sub-assemblies: this hypothesis is in good agreement with the quasi species theory proposed by Weissmann and colleagues. Another non-exclusive hypothesis relies on the ability of the prion inoculum to undergo progressive templating deformation by iterative adjustment of the host PrP structure upon oligomerization. This hypothesis also fits the model proposed by Makarava et al., which focuses on the strain adaptation. It would explain why and how a given strain, adapted to its host, is able to infect a new host, sometimes rapidly and sometimes very slowly. Finally, it is worth emphasizing that the fundamental questions addressed in this topic are supposed to bring high impact on how we understand past outbreaks of transmissible spongiform encephalopathies and potential ones. These threats are linked to hidden circulating prions (and adapting to its human host within the lymphoid organs), or to closer contacts between wild cervids and domestic ruminants (in the context of spreading of CWD in Europe).
